# Associated factors of elastic and stiffening components of pulse pressure based on 45 517 database of 24 h ambulatory blood pressure monitoring

**DOI:** 10.1038/s41371-026-01210-0

**Published:** 2026-08-26

**Authors:** Zsófia Kekk, Dorottya Pásztor, Johanna Takács, Dénes Páll, Zsófia Jósvai, Péter Torzsa, Akos Koller, Mariann Harangi, Zoltán Járai, János Nemcsik

**Affiliations:** 1 Győr-Moson-Sopron County Petz Aladár University Teaching Hospital, IV. Department of Internal Medicine, Győr, Hungary; 2 South-Buda Center Hospital St. Imre University Teaching Hospital, Department of Cardiology, Budapest, Hungary; 3 Department of Social Sciences, Semmelweis University, Budapest, Hungary; 4 Department of Medicine, University of Debrecen, Debrecen, Hungary; 5 Department of Medical Clinical Pharmacology, University of Debrecen, Debrecen, Hungary; 6 Department of Family Medicine, Semmelweis University, Budapest, Hungary; 7 Research Center for Sport Physiology, Hungarian University of Sports Science, Budapest, Hungary; 8 Departments of Morphology & Physiology and Translational Medicine, Budapest, Hungary; 9 Department of Cell and Molecular Physiology, New York Medical College, Valhalla, NY USA; 10 Division of Metabolic Diseases, Department of Internal Medicine, University of Debrecen Faculty of Medicine, Debrecen, Hungary; 11 ELKH-UD Vascular Pathophysiology Research Group, University of Debrecen, Debrecen, Hungary; 12 Institute of Health Studies, Faculty of Health Sciences, University of Debrecen, Debrecen, Hungary; 13 Section of Angiology, Városmajor Heart and Vascular Center, Semmelweis University, Budapest, Hungary

**Keywords:** Risk factors, Hypertension

## Abstract

Pulse pressure (PP), which has been proposed to be a marker of hypertension-mediated organ damage (HMOD) in elderly, can be divided into two components: elastic PP (elPP) and stiffening PP (stPP). Thus, the aim of our study was to explore the associated factors of 24 h elPP and stPP. ABPM data of 45 517 subjects (age: 56.4 ± 14.9 years, male: 20 897 (45.9%)) were collected from the Hungarian ABPM Registry between September 2020 and August 2024. Subjects’ clinical data were analyzed together with ABPM curves. elPP and stPP were calculated according to published formulas. The elPP was higher in men than in women (46.4 ± 8.2 vs 45.8 ± 9.2 mmHg, p < 0.05), whereas stPP was higher in women than in men (8.9 ± 5.8 vs 8.3 ± 5.5 mmHg, p < 0.05). elPP showed a U-shaped curve with age, which had a higher value below 40 years (47.3 ± 8 mmHg), then it decreased between 40–49 and 50–59 years (43.7 ± 7.3 and 43.9 ± 8 mmHg, respectively) and it increased again reaching 49.4 ± 9.7 mmHg above the age >70 years, while stPP progressively increased with ageing. Multiple correlates of elPP and stPP were identified based on age groups, sex, heart rate and antihypertensive medication. In conclusions, the evaluation of the associated factors of elPP and stPP and of elPP decrease in middle-aged subjects can help to better understand the pathomechanisms responsible for the development of HMOD and the role of pulse pressure in the prediction of CV outcome.

## Introduction

Globally, cardiovascular disease (CVD) is considered to be a major cause of death, with high blood pressure (BP) being one of the most significant risk factors [1, 2]. The early diagnosis of hypertension and the implementation of lifelong management strategies have been shown to be of major importance in the context of preventive healthcare [3].

In addition to systolic and diastolic blood pressure, pulse pressure (PP) is also of importance in hypertension [4]. PP is defined as the difference between systolic blood pressure (SBP) and diastolic blood pressure (DBP) [3, 5]. It is evident that the pulse wave is reflective of the arterial system’s response to ventricular ejection from the heart. Consequently, it can be deduced that vascular and cardiac factors, in conjunction with arterial wall properties, are likely to exert influence on the propagation of the pulse wave [5].

It has been demonstrated that both SBP and DBP change differently as a function of age. SBP shows a steady increase, while DBP starts to decrease after a short period of stagnation from the fifth decade onwards, resulting in an increase in PP in elderly [3, 6–8]. For women, a more pronounced increase is observed at later ages [6]. It has been established that changes in PP with ageing are associated with increased arterial wall stiffness [3, 4, 6, 9]. This is due to the remodeling of arterial vessels resulting in reduced elastin, increased collagen content, fibrotic tissue deposition and atherosclerosis [3]. Previous studies reported that an elevated PP has been demonstrated to be a contributing factor to an increased risk of CVD manifestations, encompassing coronary heart disease and heart failure [10–12]. Since 2013, hypertension treatment guidelines have identified PP levels of over 60 mmHg as a diagnostic criterion for hypertension-mediated organ damage (HMOD) in individuals aged 65 and above [3, 13, 14]. However, current European Society of Cardiology (ESC) hypertension management guidelines consider PP to be a less effective predictor in comparison to other BP parameters, and its use for risk assessment and management of hypertension is therefore advised with caution [6, 15].

**Fig. 1:**
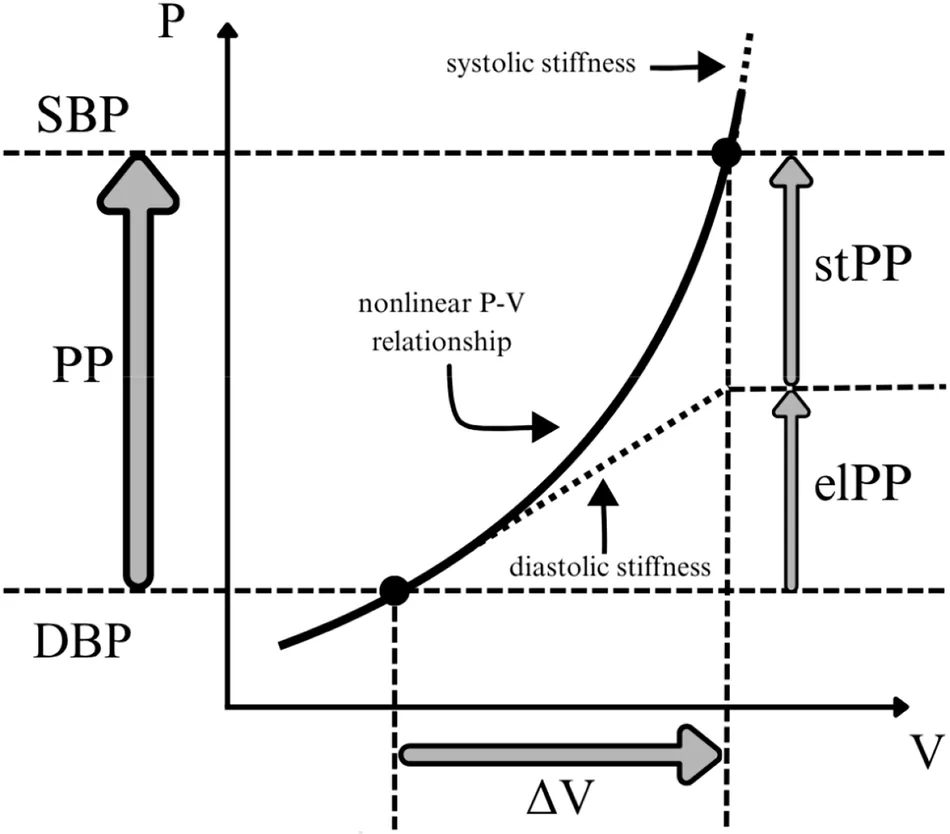
The concept of pulse pressure components. The variation of distending arterial pressure from its diastolic level to systolic level during the early systole, which is the pulse pressure (PP); the corresponding volume change (ΔV) and the stiffness change from its diastolic to systolic level. This diagram enables to view, in general, the P–V relationship during the early systole, as a superposition of a linear relationship (‘elastic’) having the slope of the diastolic stiffness and a nonlinear relationship (the original P–V relationship minus the elastic relationship). This procedure splits a given PP into the illustrated ‘elastic component’ (elPP), and a ‘stiffening component’ (stPP). Adopted from Gavish B et al. [16].

This uncertainty initiated further studies on PP, such as Gavish et al. who have developed a model-based method for decomposing PP into two components, ‘elastic’ component elPP, and a ‘stiffening’ component stPP, which are nearly independent from each other [5]. The ‘elastic’ component (elPP) is associated with arterial stiffness at the diastolic pressure, and the ‘stiffening’ component (stPP) is expressing the well-known tendency of arteries in the normal and higher pressure range, to become stiffer upon elevation of the pressure from diastole to systole [5]. Figure 1 demonstrates the concept of pulse pressure components modified after Gavish et al [16].

The fundamental premise of the model is predicated on the non-linear pressure-volume relationship that is established during systole [5, 16, 17]. This phenomenon can be attributed to the coexistence of elastic fibers and collagen in the wall of large arteries, which exhibit distinct resistance to stretch. The elastic fibers are responsible for stiffness that is unaffected by pressure (called ’elastic’), while collagen fibers, conversely, generate stiffness in response to increased stretch, with this stiffness increasing dynamically in conjunction with wall stretching (called ’stiffening’) [5, 16, 18]. elPP and stPP have been shown to be different in man and women, in different age groups and depend on the heart rate as well [5].

However, there are few studies addressing the explanatory variables of stPP and elPP in different age groups, sexes and heart rate ranges, thus no clear evidence provided recommendations of their modifications. The aim of our study was to explore the explanatory variables of 24 h elPP and stPP based on data from a national ambulatory blood pressure monitoring (ABPM) database.

## Materials and methods

The data from the Hungarian ABPM Registry were the sources of the present study. This is an ongoing multicenter, open-label, observational study, which was described in details previously [19].

In this registry, the data collection is scheduled between the years 2020 and 2035. Blood pressure and heart rate measurements were performed using the validated, oscillometric ABPM-06 (Meditech Ltd., Hungary) devices [20] on two occasions per year, at four-month intervals in family physician practices and in hypertension outpatient clinics. The primary aim of the registry was to evaluate the indications of ABPM and the characteristics of the measured population in Hungary. Adult subjects (≥ 18 years old) are involved in the registry.

The uploading of clinical data and ABPM curves were facilitated by an electronic platform. The clinical data encompass personal and family history, risk factor and comorbidity evaluation, indications for ABPM measurement, classes and number of antihypertensive medications in treated patients and the precise office BP value prior to ABPM device application. The monitors have been programmed to obtain BP measurements in 15 min intervals during the day and in 30 min intervals during the night. If the number of measurements was fewer than 7 during nighttime, fewer than 20 during daytime or fewer than 40 during the 24 h period, due to quality control, the ABPM curves were excluded from the analysis. The present analysis includes data collected between September 2020 and August 2024. There have been no repeated measurements, all ABPM curves were collected from different subjects.

About the definition of different clinical conditions we also refer or previous publication [19]. Shortly, regular coffee consumption was defined as more than 3 drinks per day. Only the presence of snoring was asked from the patient without any further details. Regular physical activity was defined as at least 3 times 30 min per week. Smoking was defined as regular cigarette smoking or with the history of smoking, there was no differentiation between traditional smoking and novel electronic devices. Obesity was defined as a body mass index ≥ 30 kg/m^2^. Hyperuricemia was defined as serum uric acid level above 360 µmol/l or with ongoing uric acid lowering therapy. Hypercholesterolemia was defined as serum total cholesterol above 5.2 mmol/l or with ongoing cholesterol-lowering therapy. Diabetes was defined as serum fasting glucose above 7 mmol/l, positive oral glucose tolerance test, HbA1c above 6.5% or with ongoing antidiabetic therapy. Hypertension was defined based on the results of ABPM in case if the 24 h average blood pressure was ≥130 mmHg systolic and/or ≥80 mmHg diastolic values.

### Calculation of pulse pressure components

The following formulae were used to quantify elPP and stPP components of PP [5]. The calculation was based on 24 h ABPM data. The five points below illustrate the above process.

i. The mean PP is be calculated from the raw ABPM data of the subject.
ii. The ratio between the standard deviations of SBP and DBP is denoted by K.
iii. The ratio stPP/elPP is expressed as (K-1)/ln(K) -1 (WIF, widening factor)
iv. elPP is given by PP/(1+ stPP/elPP)
v. stPP is PP–elPP

Where SBP is systolic blood pressure, DBP is diastolic blood pressure. K – also known as the blood pressure variability ratio or pulse stiffening ratio – represents the relative increase in arterial stiffness from diastole to systole. It corresponds to the best-fit symmetric slope of the relationship between an individual’s systolic and diastolic blood pressure values [5, 21, 22].

### Statistical analysis

Descriptive statistics were reported in mean, standard deviation and relative frequencies in the whole study sample and in subjects with or without antihypertensive medication separately. To examine the characteristics of the derived parameter K and WIF, which are crucial in the calculation of elPP and stPP, descriptive statistics, distributions, and the proportion of the upper (Q3 + 1.5xIQR) and lower (Q1-1.5xIQR) extreme values and cases with K < 1 were explored. After testing the associations between the measured variables on stPP and elPP with simple linear regression models, a final model was tested using multiple linear regression in the total sample and in subjects with or without antihypertensive medication separately. To examine the explanatory variables of stPP and elPP in different age groups (< 40, 40–49, 50–59, 60–69, ≥70 years), sexes (males, females) and different heart rate ranges (< 70 and ≥70 bpm), multiple linear regression models were used in the groups. Finally, a difference variable was calculated in the sample of 40–49 years for exploring the association between the difference and elPP using simple linear regression. The difference variable represents the difference from the mean elPP of the sample under 40 years; a positive value means a decrease in elPP, and a negative value means an increase in elPP.

The relative importance of the correlates was assessed using standardized beta coefficients complemented by unstandardized B-values with 95% confidence intervals, as well as overall model fit indices (R and R^2^). The level of significance was set at 0.05, for multiple testing adjusted p-value was used for conclusion using Bonferroni correction. Correlation was defined as strong, when beta was ≥0.2 and moderate when beta was ≥0.1. Statistical analyses were conducted using IBM SPSS Statistics for Windows, Version 27.0 (IBM Corp. Released 2017, Armonk, NY, USA).

## Results

**Fig. 2:**
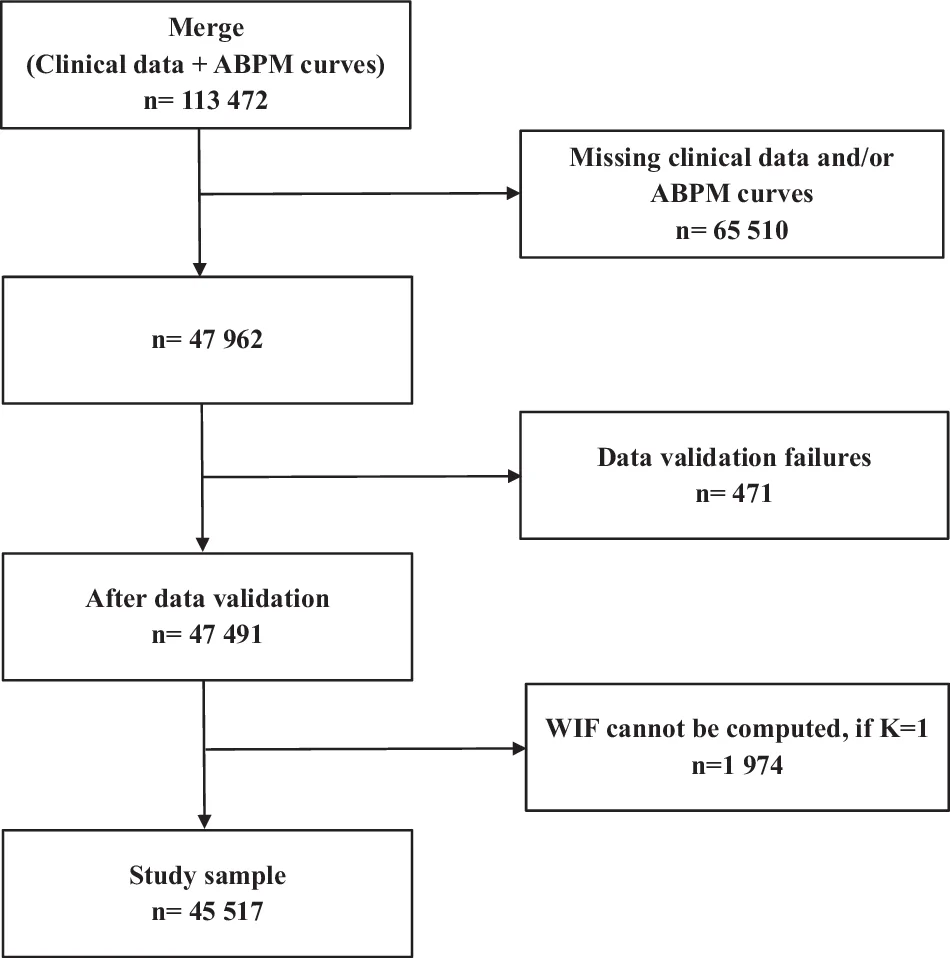
Flowchart of the study.

**Fig. 3:**
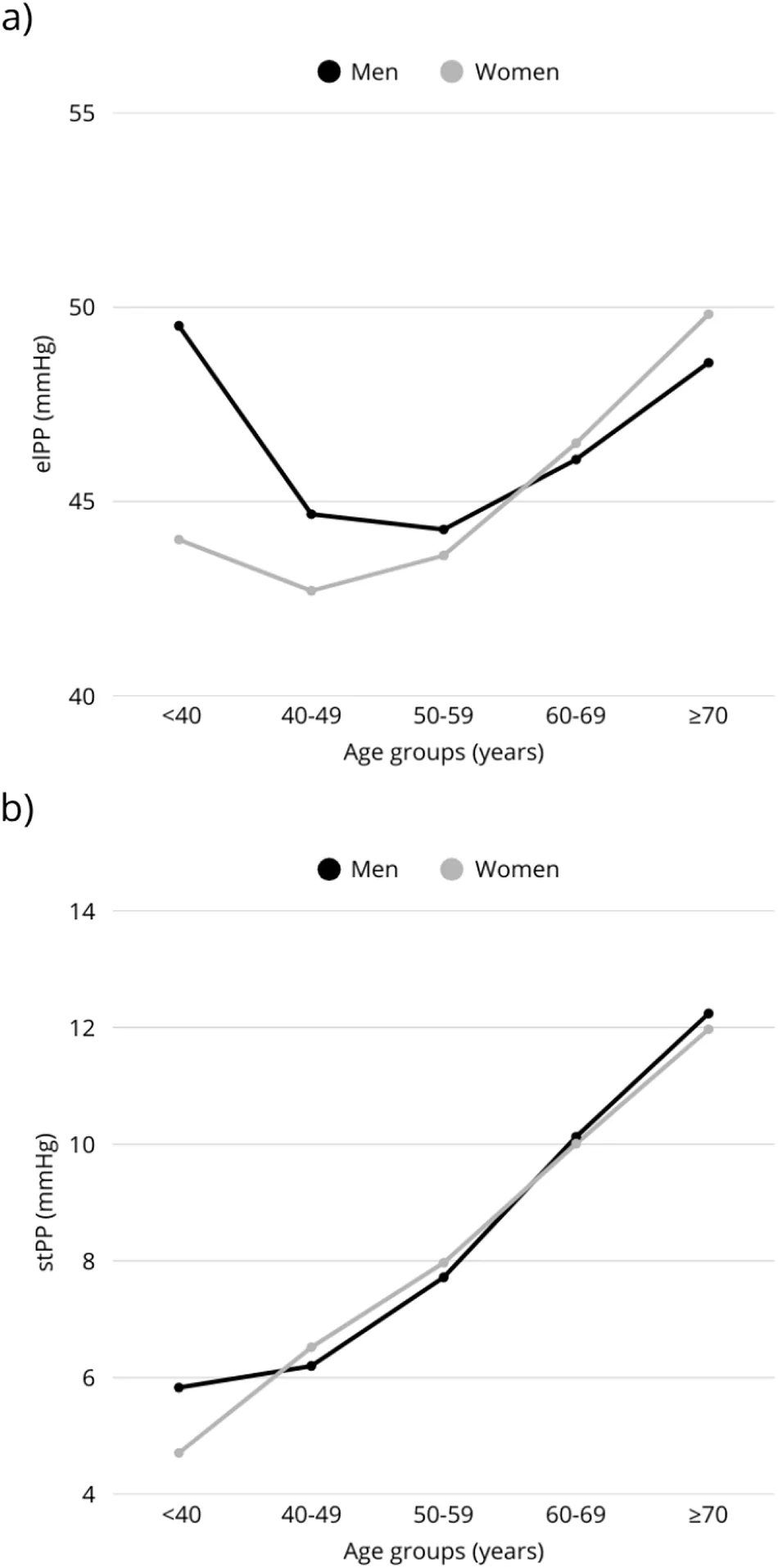
The PP components as a function of sex and age. (**a**) the elPP and (**b**) the stPP in men and women, separately. Data points illustrate the means of each PP component by 10-year age group intervals. Men are shown in black and women in gray. elPP: elastic pulse pressure; PP: pulse pressure; stPP: stiffening pulse pressure.

45 517 subjects were included into the study with ABPM curves and clinical data Fig. 2 demonstrates the flowchart of inclusion. As K was calculated 1 in 1974 cases which made impossible to calculate elPP and stPP values, these subjects had to be excluded. Descriptive clinical data of the participants in the whole study population and in subjects with or without hypertension are demonstrated in Table 1. A total of 45.9% (n = 20 897) of participants were male. The mean age of the study population was 56.40 ± 14.92 years. According to the anamnestic data, the smoking rate was 9.8%, while the coffee consumption rate was 20.1%. Lack of regular physical activity was prevalent, with 86% of study participants reporting this. The prevalence of obesity was 23.6%, diabetes 10.6%, and hypertension based on the ABPM 65.5%. The prevalence of CVD in the family history was found to be 15.6%. The mean body mass index (BMI) was 28.7 kg/m², indicating in average an overweight status of the population. The prevalence of patients with ongoing antihypertensive therapy was 72.5%. Subjects without hypertension were younger compared with the treated ones. Next, participants were divided into five age groups: <40 (13.1%), 40–49 (19.6%), 50–59 (22.5%), 60–69 (23.8%) and ≥70 (21.0%) years and were grouped according to their heart rate values (<70 or ≥70 beat/min). Supplementary Table 1 demonstrates the descriptives and the frequences of K and WIF which were crucial in the calculations of elPP and stPP in the whole study population and in subjects with or without ongoing antihypertensive medication as well. Table 2 demonstrates the elPP and stPP values in different age categories, sexes and heart rates in the whole study population and in treated and untreated patients separately. elPP showed a U-shaped curve with age and had a higher value below 40 years (47.3 ± 8 mmHg), than it decreased between 40–49 and 50–59 years (43.7 ± 7.3 and 43.9 ± 8 mmHg, respectively) and it increased again reaching 49.4 ± 9.7 mmHg above the age >70 years. The lowest point of the curve was observed in the 50–59 age group for men and in the 40–49 age group for women. The intersection of the male and female curves occurs between the 50–59 and 60–69 age groups. Prior to the intersection, males exhibited higher values in comparison to females, however this relationship is reversed after the crossing. In contrast, the stPP progressively increased with ageing. In women, the increase was more precipitous in the over-50 age groups, while in men it manifests at a younger age. The shape of the curves is illustrated in Fig. 3. Supplementary Figs. 1 and 2. demonstrates the same in untreated and treated patients, respectively. The curves did not differ markedly in the two groups. Supplementary Table 2, 3. and 4. demonstrates the explanatory variables of elPP and stPP in different sexes, and heart rates in the detailed different age groups in the total study population and in subjects with or without antihypertensive medication, respectively. Results of the whole study population and patients taking regularly antihypertensive medications showed similarities in correlates. Hypertension based on the performed ABPM was strongly correlated with elPP in both sexes, all age and heart rate categories. Obesity was strongly correlated in young and middle-aged women with elPP, while in elderly and in men the association was moderate. Diabetes showed moderate correlation with elPP in middle-aged and elderly men. In untreated subjects hypertension correlated strongly as well with elPP, but obesity was strongly correlated only in young-middle aged subjects with heart rate above 70/min. In case of stPP in the whole population and in patients with ongoing antihypertensive treatment strong correlations were found in women with hypertension in all age categories below the heart rate of 70/min, while in men it was found only in elderly. Diabetes was moderate explanatory variable of stPP only in the age between 60–69 years in both sexes below the heart rate of 70/min. In untreated subjects (Supplementary table 3.) only hypertension was clinically significant correlate in both sexes. Table 3 demonstrates the results of simple and multiple regression analysis including all variables as correlates of elPP and stPP in subjects without antihypertensive medication. Obesity has found to be a moderate explanatory variable of elPP, while hypertension based on ABPM was a strong correlate. The effect of age was not linear, a significantly lower elPP was observed in the 40–49 and 50–59 age groups compared to those under 40 years, but in older age categories the correlation turned to be positive. In the case of stPP, strong explanatory variables were the age over 60 and 70 years, while age between 50–59 years was a moderate correlate. Heart rate was negatively associated with stPP, suggesting that a higher heart rate is accompanied with a lower values. Furthermore, hypertension based on ABPM was identified as an explanatory variable of stPP in line with elPP. Table 4 demonstrates the results of simple and multiple regression analysis including all variables as correlates of elPP and stPP in subjects with ongoing antihypertensive medication. In case of elPP hypertension was a strong predictor, while obesity was moderate. The effect of age was non-linear in line with subjects without antihypertensive treatment (Table 3.), but moderate positive association was found in the age above 70 years. stPP was associated with age; while compared with the age category below 40 years, the age between 50–59 years was moderate, age between 60–69 and ≥ 70 years were strong explanatory variables. Hypertension was a strong positive correlate, while heart rate correlated negatively with stPP in a moderate way. Diabetes was moderate predictor in simple regression of both elPP and stPP, but became clinically irrelevant in multiple analysis similarly with CVD in the history in relation with stPP. The use of beta-blockers did not have impact for the PP components.

**Table 1:** Characteristics of the total study population. Results are shown in percentage (n) or mean ± standard deviation.

	Study sample (*N* = 45,517)	No antihypertensive treatment (*n* = 12,509)	Antihypertensive treatment (*n* = 33,008)
Male sex	45.9 (20,897)	49.1 (6146)	44.7 (14,751)
Age (years)	56.40 ± 14.92	48.05 ± 14.14	59.57 ± 13.95
Age categories (years)			
<40	13.1 (5944)	26.1 (3266)	8.2 (2698)
40–49	19.6 (8939)	28.4 (3546)	16.3 (5393)
50–59	22.5 (10,211)	24.4 (3051)	21.7 (7160)
60–69	23.8 (10,850)	14.4 (1087)	27.4 (9043)
≥70	21.0 (9553)	6.7 (839)	26.4 (8714)
HR (beat/min)	72.58 ± 9.51	74.80 ± 9.30	71.75 ± 9.45
HR categories (beat/min)			
<70	38.9 (17,723)	29.3 (3663)	42.6 (14,060)
≥70	61.1 (27,794)	70.7 (8846)	57.4 (18948)
Smoking	9.8 (4451)	11.1 (1388)	9.3 (3063)
Coffee consumption	20.1 (9134)	20.4 (2551)	19.9 (6583)
Snoring	28.7 (13067)	26.7 (3339)	29.5 (9728)
Lack of phys. act.	86.0 (39144)	81.5 (10201)	87.7 (28943)
Obesity	23.6 (10761)	16.9 (2116)	26.2 (8645)
CVD in the family history	15.6 (7083)	12.4 (1550)	16.8 (5533)
Diabetes mellitus	10.6 (4831)	2.9 (368)	13.5 (4463)
Hypertension (based on ABPM)	66.5 (3,0285)	71.6 (8954)	64.6 (21,331)
CVD	9.8 (4468)	1.5 (192)	13.0 (4276)
Chronic kidney disease	1.9 (859)	0.3 (37)	2.5 (822)
Hypercholesterinaemia	24.5 (11159)	12.4 (1554)	29.1 (9605)
BMI (kg/m^2^)	28.73 ± 5.16	27.81 ± 5.04	29.07 ± 5.16
SBP (mmHg)	133.58 ± 14.26	134.02 ± 13.63	133.41 ± 14.49
DBP (mmHg)	78.88 ± 10.17	82.00 ± 9.68	77.70 ± 10.10
MAP (mmHg)	97.12 ± 10.51	99.34 ± 10.30	96.28 ± 10.47
PP (mmHg)	54.69 ± 10.87	52.02 ± 9.07	55.70 ± 11.32
elPP (mmHg)	46.05 ± 8.73	45.04 ± 7.92	46.43 ± 8.99
stPP (mmHg)	8.65 ± 5.71	6.98 ± 4.65	9.28 ± 5.94

*BMI* body mass index, *CVD* cardiovascular disease, *DBP* diastolic blood pressure, *elPP* elastic pulse pressure, *HR* heart rate, *Lack of phys. act*. lack of regular physical activity, *MAP* mean arterial pressure, *PP* pulse pressure, *SBP* systolic blood pressure, *stPP* stiffening pulse pressure

**Table 2:** The elPP and stPP values by sexes, different age categories and heart rates. Results are shown in mean (standard deviation).

Sex	Age	HR	Study sample		No antihypertensive treatment		Antihypertensive treatment	
			elPP	stPP	elPP	stPP	elPP	stPP
Male	<40	<70	50.34 (7.63)	6.25 (4.84)	50.73 (7.78)	6.09 (4.91)	49.87 (7.43)	6.46 (4.76)
		≥70	49.19 (7.46)	5.66 (4.40)	49.06 (7.43)	5.37 (4.23)	49.35 (7.49)	6.02 (4.57)
	40–49	<70	44.40 (6.53)	6.26 (4.07)	43.83 (6.51)	6.13 (3.97)	44.78 (6.52)	6.35 (4.13)
		≥70	44.78 (7.09)	6.18 (4.02)	44.22 (6.68)	5.93 (3.75)	45.13 (7.31)	6.33 (4.18)
	50–59	<70	44.39 (7.55)	8.17 (4.86)	43.98 (6.60)	7.82 (4.53)	44.55 (7.87)	8.30 (4.97)
		≥70	44.22 (7.56)	7.48 (4.40)	43.69 (7.32)	7.18 (4.24)	44.43 (7.64)	7.59 (4.46)
	60–69	<70	46.26 (8.26)	10.80 (5.64)	45.71 (7.33)	9.74 (4.92)	46.36 (8.42)	10.99 (5.74)
		≥70	45.92 (5.58)	9.51 (5.35)	45.24 (8.01)	9.01 (4.57)	46.07 (8.69)	9.62 (5.49)
	≥70	<70	48.96 (9.14)	13.15 (6.62)	47.67 (8.69)	11.26 (5.99)	49.09 (9.18)	13.34 (6.65)
		≥70	47.89 (9.27)	10.64 (5.77)	48.38 (9.11)	10.02 (5.32)	47.82 (9.29)	10.73 (5.83)
Female	<40	<70	43.14 (6.90)	4.41 (3.67)	42.15 (6.93)	4.04 (3.52)	44.26 (6.70)	4.83 (3.82)
		≥70	44.16 (7.50)	4.75 (3.91)	44.09 (7.36)	4.63 (3.78)	44.25 (7.67)	4.90 (4.07)
	40–49	<70	42.01 (6.95)	7.02 (3.97)	41.73 (6.94)	6.91 (3.55)	42.17 (6.95)	7.09 (4.19)
		≥70	42.88 (7.65)	6.39 (4.06)	42.65 (7.30)	6.18 (3.86)	43.05 (7.89)	6.55 (4.19)
	50–59	<70	42.75 (7.86)	8.40 (4.62)	41.65 (6.99)	8.46 (3.91)	43.16 (8.13)	8.38 (4.86)
		≥70	43.99 (8.53)	7.78 (4.79)	43.66 (8.06)	7.34 (4.33)	44.16 (8.76)	8.02 (5.00)
	60–69	<70	46.14 (9.10)	10.88 (5.93)	45.25 (8.24)	10.19 (5.49)	46.28 (9.23)	10.99 (5.99)
		≥70	46.78 (9.35)	9.30 (5.54)	45.72 (8.36)	8.59 (4.87)	47.04 (9.55)	9.47 (5.68)
	≥70	<70	49.76 (9.87)	12.85 (6.72)	49.62 (8.83)	11.21 (5.56)	49.77 (9.93)	12.96 (6.78)
		≥70	49.91 (9.94)	10.73 (6.08)	48.65 (9.10)	9.98 (4.91)	50.05 (10.03)	10.82 (6.19)

*elPP* elastic pulse pressure, *HR* heart rate, *stPP* stiffening pulse pressure.

**Table 3:** The results of simple and multiple regression analysis including all variables for the prediction of elPP and stPP in subjects without antihypertensive treatment.

elPP	Simple, R^2^ = 0.198							Multiple, R^2^ = 0.197						
	B	SE	95% CI		Beta	t	p	B	SE	95% CI		Beta	t	p
male sex	***2.22***	***0.14***	***1.95***	***2.50***	***0.140***	***15.849***	***<0.001***	1.06	0.13	0.79	1.32	0.067	7.876	<0.001
age. ref: <40 yrs														
40–49 yrs	−**4.02**	**0.19**	−**4.38**	−**3.65**	−**0.228**	−**21.542**	**<0.001**	−**4.48**	**0.18**	−**4.82**	−**4.13**	−**0.255**	−**25.517**	**<0.001**
50–59 yrs	−**3.88**	**0.19**	−**4.26**	−**3.50**	−**0.210**	−**20.028**	**<0.001**	−**4.15**	**0.19**	−**4.52**	−**3.79**	−**0.225**	−**22.427**	**<0.001**
60–69 yrs	−1.79	0.23	−2.23	−1.35	−0.079	−7.950	<0.001	−1.57	0.22	−1.99	−1.14	−0.070	−7.238	<0.001
≥70 yrs	1.33	0.30	0.75	1.91	0.042	4.476	<0.001	1.97	0.28	1.42	2.53	0.062	6.936	<0.001
heart rate	0.00	0.01	−0.01	0.02	0.004	0.471	0.638	−0.04	0.01	−0.06	−0.03	−0.048	−5.590	<0.001
smoking	−0.06	0.23	−0.50	0.39	−0.002	−0.253	0.800	−0.01	0.21	−0.42	0.39	−0.001	−0.064	0.949
coffee consumption	−0.38	0.18	−0.72	−0.03	−0.019	−2.153	0.031	−0.26	0.16	−0.58	0.05	−0.013	−1.638	0.101
snoring	0.73	0.16	0.41	1.04	0.041	4.534	<0.001	0.25	0.15	−0.04	0.55	0.014	1.670	0.095
lack of phys. act.	0.37	0.18	0.01	0.73	0.018	2.031	0.042	0.12	0.17	−0.20	0.45	0.006	0.738	0.460
obesity	***3.75***	***0.19***	***3.39***	***4.12***	***0.178***	***20.184***	***<0.001***	***3.37***	***0.18***	***3.03***	***3.72***	***0.160***	***19.157***	***<0.001***
family history	−0.46	0.22	−0.88	−0.04	−0.019	−2.129	0.033	−0.41	0.20	−0.80	−0.03	−0.017	−2.091	0.037
diabetes	2.57	0.42	1.75	3.39	0.055	6.145	<0.001	1.61	0.38	0.86	2.36	0.034	4.218	<0.001
hypertension	**5.17**	**0.15**	**4.88**	**5.47**	**0.294**	**34.454**	**<0.001**	**5.30**	**0.15**	**5.01**	**5.59**	**0.302**	**35.803**	**<0.001**
CVD	0.82	0.58	−0.31	1.95	0.013	1.422	0.155	0.37	0.52	−0.66	1.39	0.006	0.705	0.481
chronic kidney disease	−1.70	1.30	−4.26	0.85	−0.012	−1.306	0.192	−2.21	1.17	−4.51	0.09	−0.015	−1.886	0.059
hypercholesterinaemia	−0.65	0.21	−1.07	−0.23	−0.027	−3.023	0.003	−0.94	0.20	−1.33	−0.55	−0.039	−4.712	<0.001

stPP	Simple, R^2^ = 0.161							Multiple, R^2^ = 0.160						
	B	SE	95% CI		Beta	t	p	B	SE	95% CI		Beta	t	p
male sex	−0.28	0.08	−0.44	−0.11	−0.030	−3.331	0.001	−0.30	0.08	−0.46	−0.14	−0.032	−3.730	<0.001
age. ref: <40 yrs														
40–49 yrs	0.98	0.11	0.77	1.19	0.095	9.287	<0.001	0.70	0.11	0.50	0.91	0.068	6.692	<0.001
50–59 yrs	**2.35**	**0.11**	**2.14**	**2.57**	**0.217**	**21.519**	**<0.001**	***1.99***	***0.11***	***1.77***	***2.21***	***0.184***	***17.890***	***<0.001***
60–69 yrs	**4.07**	**0.13**	**3.82**	**4.32**	**0.308**	**31.977**	**<0.001**	**3.69**	**0.13**	**3.44**	**3.95**	**0.279**	**28.380**	**<0.001**
≥70 yrs	**5.44**	**0.17**	**5.11**	**5.77**	**0.293**	**32.348**	**<0.001**	**5.04**	**0.17**	**4.71**	**5.38**	**0.271**	**29.507**	**<0.001**
heart rate	−***0.08***	***0.00***	−***0.09***	−***0.07***	−***0.162***	−***18.346***	***<0.001***	−***0.06***	***0.00***	−***0.07***	−***0.05***	−***0.126***	−***14.245***	***<0.001***
smoking	−0.71	0.13	−0.97	−0.45	−0.048	−5.359	<0.001	−0.01	0.12	−0.25	0.24	<0.001	−0.058	0.953
coffee consumption	−0.23	0.10	−0.44	−0.03	−0.020	−2.274	0.023	−0.09	0.10	−0.28	0.10	−0.008	−0.942	0.346
snoring	0.18	0.09	0.00	0.37	0.017	1.925	0.054	0.15	0.09	−0.02	0.33	0.015	1.708	0.088
lack of phys. act.	0.27	0.11	0.06	0.48	0.023	2.525	0.012	0.14	0.10	−0.06	0.34	0.012	1.381	0.167
obesity	0.07	0.11	−0.15	0.29	0.006	0.634	0.526	0.24	0.11	0.04	0.45	0.020	2.295	0.022
family history	−0.48	0.13	−0.72	−0.23	−0.034	−3.783	<0.001	−0.28	0.12	−0.51	−0.05	−0.020	−2.351	0.019
diabetes	1.57	0.25	1.09	2.05	0.057	6.388	<0.001	0.89	0.23	0.44	1.34	0.032	3.886	<0.001
hypertension	***1.24***	***0.09***	***1.06***	***1.42***	***0.121***	***13.594***	***<0.001***	***1.63***	***0.09***	***1.46***	***1.80***	***0.158***	***18.363***	***<0.001***
CVD	1.54	0.34	0.87	2.20	0.041	4.549	<0.001	0.34	0.31	−0.28	0.95	0.009	1.078	0.281
chronic kidney disease	1.68	0.77	0.18	3.18	0.020	2.195	0.028	0.21	0.70	−1.17	1.59	0.002	0.300	0.764
hypercholesterinaemia	0.59	0.13	0.34	0.83	0.042	4.649	<0.001	−0.13	0.12	−0.36	0.10	−0.009	−1.102	0.271

*CVD* cardiovascular disease, *elPP* elastic pulse pressure, *lack of phys. act*. lack of regular physical activity, *stPP* stiffening pulse pressure; 95% CI: 95% confidence interval for B, lower and upper bound; Bold: clinically significant strong predictor; Bold in italics: clinically significant moderate predictor.

**Table 4:** The results of simple and multiple regression analysis including all variables for the prediction of elPP and stPP in subjects with ongoing antihypertensive treatment.

elPP	Simple, R^2^ = 0.270							Multiple, R^2^ = 0.270						
	B	SE	95% CI		Beta	t	p	B	SE	95% CI		Beta	t	p
male sex	0.01	0.10	−0.19	0.20	0.000	0.071	0.943	−0.64	0.09	−0.82	−0.47	−0.036	−7.274	0.000
age. ref: <40 yrs														
40–49 yrs	−***3.44***	***0.21***	−***3.85***	−***3.04***	−***0.142***	−***16.725***	***<0.001***	−***4.05***	***0.18***	−***4.41***	−***3.70***	−***0.167***	−***22.261***	***<0.001***
50–59 yrs	−***3.27***	***0.20***	−***3.66***	−***2.89***	−***0.150***	−***16.594***	***<0.001***	−***3.70***	***0.18***	−***4.04***	−***3.35***	−***0.169***	−***20.983***	***<0.001***
60–69 yrs	−0.93	0.19	−1.31	−0.56	−0.046	−4.868	<0.001	−0.93	0.18	−1.27	−0.58	−0.046	−5.288	<0.001
≥70 yrs	2.04	0.19	1.66	2.42	0.100	10.605	<0.001	***2.20***	***0.18***	***1.85***	***2.56***	***0.108***	***12.060***	***<0.001***
heart rate	−0.05	0.00	−0.06	−0.04	−0.052	−9.495	<0.001	−0.07	0.00	−0.08	−0.06	−0.076	−14.851	<0.001
smoking	−0.78	0.17	−1.12	−0.45	−0.025	−4.592	<0.001	0.38	0.15	0.09	0.67	0.012	2.532	0.011
coffee consumption	−0.19	0.12	−0.43	0.05	−0.009	−1.548	0.122	0.10	0.11	−0.11	0.31	0.005	0.956	0.339
snoring	0.72	0.11	0.51	0.93	0.036	6.627	<0.001	0.35	0.10	0.16	0.54	0.018	3.565	<0.001
lack of phys. act.	1.43	0.15	1.14	1.73	0.052	9.518	<0.001	0.66	0.13	0.40	0.91	0.024	5.023	<0.001
obesity	***3.76***	***0.11***	***3.54***	***3.98***	***0.184***	***33.988***	***<0.001***	***2.88***	***0.10***	***2.69***	***3.08***	***0.141***	***28.502***	***<0.001***
family history	−0.38	0.13	−0.64	−0.12	−0.016	−2.835	0.005	−0.31	0.12	−0.53	−0.08	−0.013	−2.662	0.008
diabetes	***3.88***	***0.14***	***3.60***	***4.16***	***0.148***	***27.098***	***<0.001***	2.03	0.13	1.77	2.29	0.077	15.555	<0.001
hypertension	**7.28**	**0.10**	**7.09**	**7.46**	**0.387**	**76.217**	**<0.001**	**7.87**	**0.09**	**7.69**	**8.05**	**0.418**	**85.422**	***<0.001***
CVD	1.58	0.15	1.30	1.87	0.059	10.765	<0.001	0.05	0.13	−0.22	0.31	0.002	0.346	0.730
chronic kidney disease	3.87	0.32	3.24	4.49	0.067	12.195	<0.001	1.28	0.28	0.74	1.82	0.022	4.640	<0.001
hypercholesterinaemia	0.66	0.11	0.45	0.88	0.033	6.074	<0.001	−0.36	0.10	−0.56	−0.17	−0.018	−3.674	<0.001
beta blockers	0.11	0.10	−0.09	0.31	0.006	1.044	0.297	−0.16	0.09	−0.34	0.02	−0.009	−1.789	0.074

stPP	Simple, R^2^ = 0.217							Multiple, R^2^ = 0.217						
	B	SE	95% CI		Beta	t	p	B	SE	95% CI		Beta	t	p
male sex	−0.62	0.07	−0.75	−0.49	−0.052	−9.465	<0.001	−0.38	0.06	−0.50	−0.27	−0.032	−6.353	<0.001
age. ref: <40 yrs														
40–49 yrs	0.85	0.13	0.60	1.10	0.053	6.582	<0.001	0.51	0.12	0.27	0.75	0.032	4.094	<0.001
50–59 yrs	***2.34***	***0.12***	***2.10***	***2.59***	***0.163***	***18.918***	***<0.001***	***1.86***	***0.12***	***1.63***	***2.10***	***0.129***	***15.462***	***<0.001***
60–69 yrs	**4.57**	**0.12**	**4.34**	**4.81**	**0.343**	**38.017**	**<0.001**	**3.88**	**0.12**	**3.64**	**4.11**	**0.291**	**32.359**	**<0.001**
≥70 yrs	**6.56**	**0.12**	**6.32**	**6.79**	**0.487**	**54.258**	**<0.001**	**5.51**	**0.13**	**5.26**	**5.75**	**0.408**	**44.032**	**<0.001**
heart rate	−**0.15**	**0.00**	−**0.16**	−**0.14**	−**0.240**	−**44.908**	**<0.001**	−***0.11***	***0.00***	−***0.11***	−***0.10***	−***0.171***	−***32.313***	***<0.001***
smoking	−1.05	0.11	−1.27	−0.83	−0.051	−9.362	<0.001	0.38	0.10	0.18	0.58	0.019	3.743	<0.001
coffee consumption	−0.80	0.08	−0.96	−0.64	−0.054	−9.777	<0.001	−0.29	0.07	−0.43	−0.14	−0.019	−3.876	<0.001
snoring	−0.24	0.07	−0.38	−0.10	−0.018	−3.314	0.001	−0.01	0.07	−0.14	0.12	−0.001	−0.103	0.918
lack of phys. act.	0.92	0.10	0.72	1.11	0.051	9.242	<0.001	0.38	0.09	0.20	0.55	0.021	4.198	<0.001
obesity	0.32	0.07	0.17	0.46	0.023	4.248	<0.001	0.22	0.07	0.09	0.36	0.016	3.196	0.001
family history	−0.71	0.09	−0.88	−0.53	−0.044	−8.074	<0.001	−0.26	0.08	−0.42	−0.11	−0.017	−3.341	0.001
diabetes	***2.33***	***0.09***	***2.15***	***2.52***	***0.134***	***24.625***	***<0.001***	1.14	0.09	0.96	1.31	0.066	12.754	<0.001
hypertension	***1.69***	***0.07***	***1.56***	***1.82***	***0.136***	***24.972***	***<0.001***	**2.70**	**0.06**	**2.58**	**2.82**	**0.217**	**42.830**	**<0.001**
CVD	***2.42***	***0.10***	***2.23***	***2.60***	***0.137***	***25.044***	***<0.001***	0.54	0.09	0.36	0.72	0.031	5.860	<0.001
chronic kidney disease	3.01	0.21	2.60	3.42	0.079	14.405	<0.001	0.94	0.19	0.57	1.31	0.025	4.971	<0.001
hypercholesterinaemia	1.06	0.07	0.92	1.20	0.081	14.811	<0.001	−0.04	0.07	−0.17	0.10	−0.003	−0.553	0.581
beta blockers	0.68	0.07	0.55	0.81	0.055	9.982	<0.001	0.09	0.06	−0.03	0.21	0.007	1.419	0.156

*CVD* cardiovascular disease, *elPP* elastic pulse pressure, *lack of phys. act*. lack of regular physical activity, *stPP* stiffening pulse pressure; 95% CI: 95% confidence interval for B, lower and upper bound; Bold: clinically significant strong predictor; Bold in italics: clinically significant moderate predictor.

Finally, we analyzed the correlates of the decrease of elPP between the age of 40–49 years compared with the average value of elPP before the age of 40 year. Table 5 demonstrates the results. Strong predictors were obesity and hypertension, while moderate ones were male sex and snoring. However it also has to be mentioned that elPP in this age category decreased only in 73.9% (n = 6605) of the subjects, while in 26.1% (n = 2334) it remained equal or increased.

**Table 5:** The results of simple regression analysis, including all variables for the association between the difference* and elPP in the sample of 40–49 years (*n* = 8939).

	B	SE	95% CI		Beta	t	p	R	R^2^
male sex	−***1.973***	***0.15***	−***2.27***	−***1.67***	−***0.135***	−***12.919***	***<0.001***	***0.14***	***0.02***
heart rate	−0.037	0.01	−0.05	−0.02	−0.045	−4.270	<0.001	0.05	0.00
smoking	−0.303	0.23	−0.75	0.15	−0.014	−1.319	0.187	0.01	0.00
coffee consumption	−0.270	0.18	−0.63	0.09	−0.016	−1.484	0.138	0.02	0.00
snoring	−***1.738***	***0.16***	−***2.06***	−***1.42***	−***0.112***	−***10.608***	***<0.001***	***0.11***	***0.01***
do not exercise	−0.352	0.21	−0.76	0.05	−0.018	−1.709	0.087	0.02	0.00
obesity	−**4.240**	**0.17**	−**4.58**	−**3.90**	−**0.252**	−**24.593**	**<0.001**	**0.25**	**0.06**
family history	−0.247	0.20	−0.64	0.15	−0.013	−1.232	0.218	0.01	0.00
diabetes	−2.834	0.35	−3.52	−2.14	−0.085	−8.042	<0.001	0.09	0.01
hypertension	−**4.685**	**0.17**	−**5.02**	−**4.35**	−**0.275**	−**27.074**	**<0.001**	**0.28**	**0.08**
CVD	0.106	0.49	−0.86	1.07	0.002	0.216	0.829	0.00	0.00
chronic kidney disease	0.013	0.87	−1.69	1.71	0.000	0.015	0.988	0.00	0.00
hypercholesterinaemia	−0.130	0.21	−0.54	0.28	−0.007	−0.618	0.537	0.01	0.00

^*^The difference from the mean elPP of the sample under 40 years (M = 47.3512); CVD: cardiovascular disease; elPP: elastic pulse pressure; 95% CI: 95% confidence interval for B, lower and upper bound; Bold: clinically significant strong association; Bold in italics: clinically significant moderate association.

## Discussion

The main findings of our present study are that 1) the PP components changes as function of age with different pattern in case of elPP and stPP; 2) sexual differences are present in the age-dependent changes of elPP; 3) differences were found in the explanatory variables of both PP components depending on the treatment of hypertension; 4) besides age, hypertension based on ABPM and obesity were the most important correlates of elPP, while in case of stPP heart rate and hypertension were clinically relevant explanatory variables; 5) the decrease of elPP between the age of 40–49 years correlated strongly with obesity and hypertension and moderately with male sex and snoring.

In previous studies on PP components, Gavish et al. [5, 16] demonstrated several results which are in line with the findings of our study. In their research, elPP also showed a curved line, with lowest values around 50 years for men and 40 years for women, with the lines intersecting between 50 and 60 years of age and displaying a similar trend to our results both before and after the intersection point. Furthermore, a linear increase in stPP with age was also observed. Their study revealed that all PP variables exhibited elevated values in older age groups, individuals with elevated mean arterial blood pressure, in women, in individuals with a lower heart rate, and in individuals with SBP dipping [5]. The levels of elPP, stPP and PP were elevated in patients with treated diabetes. Positive correlation was observed between higher body mass index (BMI) and elevated elPP [5]. Thus, our findings led us to similar conclusions with regarding to predictors of PP components. However, our database enabled more detailed analysis and additional explanatory variables, like lack of regular physical activity, snoring or hypercholesterinemia were also identified. We also had the opportunity to analyze the data based on the presence or absence of antihypertensive treatment. In treated hypertensive patients age ≥ 70 years was and additional explanatory variable in case of elPP, while in simple regression diabetes and CVD were correlates of stPP. The use of beta-blockers did not have impact.

The elPP values demonstrate a U-shaped curve, which may indicate that early changes in arterial wall structure may already are present at a young age, while a transitional balance is established in middle age, followed by further structural changes in the arterial wall associated with the process of aging. In contrast, stPP values increased gradually and continuously with age, which is consistent with the age-related process of arterial stiffening described in the literature [5, 16, 23]. Connected to the U-shape curve of elPP our new hypothesis might resolve a contradiction about the predictive value of pulse pressure in age below 60 years. The decrease of elPP between the age of 40–49 years compared with the average value below 40 years (due to e.g. endothelial dysfunction [24]) precedes the progression of stPP which is remarkable from the age of 50 years. This decrease in elPP was strongly correlated with obesity and hypertension and moderately with male sex and snoring, which are known CV risk factors. We presume that when in this age category in case of obesity diastolic hypertension develop, it leads to the decrease of pulse pressure, which in this age is mostly elastic PP. We hypothesize, that between the age of 40–49 years the decrease of pulse pressure could be predictive for CV events. Our hypothesis is confirmed by the study of Gavish B et al [16], where in the age between 40–50 years higher elPP was associated with lower CV mortality with borderline significance (hazard ratio: 0.42 (0.17–1.01), p = 0.054). So presumably a future target in this age category can be the preservation of elPP with the treatment of obesity and diastolic hypertension, a hypothesis, which is needed to be confirmed by prospective studies.

The question arises if the progression of arterial stiffening is a cause or a consequence of hypertension? Arterial stiffening and hypertension are strongly interrelated as arterial stiffness has impact on pulse pressure (as our study also demonstrates) and increasing blood pressure leads to the progression of arterial stiffening [25]. The Framingham Offspring study and the Baltimore Longitudinal Study of Ageing clearly demonstrated that arterial stiffening (measured with pulse wave velocity, PWV) is a precursor of incident hypertension [26, 27]. A meta-analysis also confirmed that higher PWV independently from systolic and diastolic blood pressure predicts incident hypertension [28]. In contrast, incident hypertension seems not to predict the progression of arterial stiffening [26, 27, 29–31]. However, the presence of bidirectional relation is supported by the results of a twin study, in which the annual progression of PWV was associated with mean arterial pressure (MAP) progression and MAP progression was associated with prior PWV [32]. These findings suggest that proper treatment of hypertension may prevent a cycle of arterial stiffening and hypertension and presumably the increase of stPP.

Gender differences were also observed in our study. Pulse pressure values exhibited a more pronounced increase in women, particularly those over the age of 50 years, compared to men. This is likely attributable to the decline in estrogen levels that occurs following menopause, as well as to the distinct vascular adaptation mechanisms exhibited by women [33, 34]. In men the rise in pulse pressure commenced at an earlier age, during middle age, which is consistent with the previously documented elevated cardiovascular risk in this age category, compared to women [3].

The relationship between heart rate and pulse pressure proved to be particularly significant in the case of stPP, given that higher heart rate values (≥70/min) were associated with significantly lower stPP in both untreated subjects and patients with ongoing antihypertensive therapy. This finding may be due to the fact that as heart rate increases, it reduces the diastolic time, which in turn affects central pressure equalization. In contrast, the relationship with heart rate was not significant in the case of elPP, which may indicate that other factors, such as the elasticity of the vessel wall or blood pressure wave propagation conditions, may have a greater impact on elPP.

Our findings can have important notes for the daily clinical practice. We demonstrated the dynamic changes of risk factors of detrimental pulse pressure component progression with ageing and in the two sexes. In this relation blood pressure control and the reduction of body weight also have outmost importance. In light of our findings, specific areas of focused preventive strategies can be developed in different age groups, to reduce target organ damage and improve cardiovascular outcome. It may include optimal blood pressure control, healthy eating habits, increased physical activity and perhaps pharmacotherapy to reduce obesity. However, to clarify the impact of these interventions on PP components requires prospective studies to confirm this hypothesis.

The present study has some limitations. Due to the nature of the study, namely that it was conducted using a cross-sectional analysis, it was not possible to evaluate causal relationships between the studied parameters. Furthermore, the medical histories of the subjects were based on the reports provided by the attending physicians, which may have contained inaccuracies.

In conclusions, the present study identified risk factors which modulate the elastic and the stiff components of pulse pressure. This knowledge may help to better understand the pathophysiological mechanisms responsible for the development of HMOD. The decrease of elPP between before the increase of stPP in the age of 40–49 years together with its correlates (obesity and hypertension) might explain the contradictions of the predictive value of pulse pressure in age below 60 years. The incorporation of elPP and stPP into routine ABPM analyses may contribute to the development of preventive patient care and to the development of better, more personalized interventions.

## Summary

### What is known about topic

- Pulse pressure (PP) can be separated to elastic (elPP) and stiff (stPP) components.
- The separated analysis of elPP and stPP might resolve the contradictions of the prognostic value of PP in younger ages.

### What this study adds

- Multiplex variables were studied to evaluate correlates of elPP and stPP in different age categories, sexes, heart rate categories and based on the use of antihypertensive medication.
- The decrease of elPP between before the increase of stPP in the age of 40–49 years was strongly associated with obesity and hypertension and moderately with male sex and snoring, an observation which might explain the contradictions of the predictive value of pulse pressure in age below 60 years.

## Supplementary information

Supplementary Table 1.

Supplementary Table 2.

Supplementary Table 3.

Supplementary Table 4.

Supplementary Figure 1.

Supplementary Figure 2.

Data file 1

## Data Availability

The dataset supporting the conclusions of this article is included within the article in an additional excel file.
