# Supplementary material for: Associated factors of elastic and stiffening components of pulse pressure based on 45 517 database of 24 h ambulatory blood pressure monitoring
Source: J Hum Hypertens. 2026 Aug 26;40(11):855–64. doi: 10.1038/s41371-026-01210-0 (PMC13747817; doi:10.1038/s41371-026-01210-0)
Supplement: Supplementary file 1 [file 41371_2026_1210_MOESM1_ESM.docx]

Supplementary Table 1. Descriptive statistics of K and WIF

|  | Study sample, N=45517 | |
| --- | --- | --- |
|  | K | WIF |
| mean (SE) | 1.42 (0.00) | 0.19 (0.00) |
| 95% CI for mean, lower;upper | 1.412;1.418 | 0.190;0.193 |
| 5% trimmed mean | 1.40 | 0.19 |
| median | 1.36 | 0.17 |
| 1st quartile | 1.22 | 0.11 |
| 3rd quartile | 1.55 | 0.26 |
| variance | 0.08 | 0.02 |
| std. deviation | 0.28 | 0.12 |
| minimum | 0.67 | -0.18 |
| maximum | 4.14 | 1.21 |
| range | 3.48 | 1.39 |
| interquartile range | 0.33 | 0.15 |
| skewness (SE) | 1.12 (0.01) | 0.88 (0.01) |
| kurtosis (SE) | 2.57 (0.02) | 1.65 (0.02) |
| upper extreme values, n (%) | 1266 (0.03) | 1064 (0.02) |
| lower extreme values, n (%) | 19 (0.0004) | 34 (0.001) |
| K<1, n (%) | 1114 (0.02) |  |

|  | No antihypertensive treatment, n=12509 | |
| --- | --- | --- |
|  | K | WIF |
| mean (SE) | 1.34 (0.00) | 0.16 (0.00) |
| 95% CI for mean, lower;upper | 1.338;1.346 | 0.158;0.162 |
| 5% trimmed mean | 1.33 | 0.16 |
| median | 1.31 | 0.15 |
| 1st quartile | 1.18 | 0.09 |
| 3rd quartile | 1.46 | 0.22 |
| variance | 0.06 | 0.01 |
| std. deviation | 0.23 | 0.10 |
| minimum | 0.67 | -0.18 |
| maximum | 3.00 | 0.82 |
| range | 2.33 | 1.00 |
| interquartile range | 0.28 | 0.13 |
| skewness (SE) | 0.96 (0.02) | 0.73 (0.02) |
| kurtosis (SE) | 2.10 (0.04) | 1.45 (0.04) |
| upper extreme values, n (%) | 334 (0.03) | 243 (0.02) |
| lower extreme values, n (%) | 10 (0.001) | 15 (0.001) |
| K<1, n (%) | 432 (0.03) |  |

|  | Antihypertensive treatment, n=33008 | |
| --- | --- | --- |
|  | K | WIF |
| mean (SE) | 1.44 (0.00) | 0.20 (0.00) |
| 95% CI for mean, lower;upper | 1.439;1.446 | 0.203;0.205 |
| 5% trimmed mean | 1.425 | 0.198 |
| median | 1.40 | 0.19 |
| 1st quartile | 1.23 | 0.11 |
| 3rd quartile | 1.60 | 0.28 |
| variance | 0.08 | 0.02 |
| std. deviation | 0.29 | 0.13 |
| minimum | 0.67 | -0.18 |
| maximum | 4.14 | 1.21 |
| range | 3.48 | 1.39 |
| interquartile range | 0.37 | 0.17 |
| skewness (SE) | 1.10 (0.01) | 0.86 (0.01) |
| kurtosis (SE) | 2.42 (0.03) | 1.52 (0.03) |
| upper extreme values, n (%) | 728 (0.02) | 464 (0.01) |
| lower extreme values, n (%) | 2 (0.0001) | 3 (0.0001) |
| K<1, n (%) | 681 (0.01) |  |
