# Supplementary material for: Associated factors of elastic and stiffening components of pulse pressure based on 45 517 database of 24 h ambulatory blood pressure monitoring
Source: J Hum Hypertens. 2026 Aug 26;40(11):855–64. doi: 10.1038/s41371-026-01210-0 (PMC13747817; doi:10.1038/s41371-026-01210-0)
Supplement: Supplementary file 2 [file 41371_2026_1210_MOESM2_ESM.docx]

**Supplementary table 2.** The explanatory variables of elPP and stPP in different sexes and heart rates in the detailed different age categories in the whole study population.

| **FEMALE, elPP** | | | | | | | | | | | | | |
| --- | --- | --- | --- | --- | --- | --- | --- | --- | --- | --- | --- | --- | --- |
| **AGE** | **HR** | smoking | coffee consump-tion | snoring | lack of phys. activity | obesity | family history | diabetes | hyper-tension | CVD | chronic kidney disease | hyper-choleste-rinaemia | R^2^ |
| **<40** | **<70** |  |  |  |  | **0,225** |  |  | ***0,127*** |  |  |  | 0,113 |
|  | **≥70** |  |  |  | 0,053 | **0,325** |  |  | **0,217** |  |  |  | 0,188 |
| **40-49** | **<70** |  |  |  |  | **0,245** |  |  | **0,283** |  |  |  | 0,170 |
|  | **≥70** |  |  | 0,068 |  | **0,269** |  | 0,050 | **0,254** |  |  |  | 0,176 |
| **50-59** | **<70** |  |  |  |  | **0,224** |  |  | **0,404** |  |  |  | 0,262 |
|  | **≥70** |  |  |  | 0,640 | **0,217** |  | 0,094 | **0,317** |  |  | -0,059 | 0,199 |
| **60-69** | **<70** |  |  | 0,055 |  | ***0,128*** |  | 0,085 | **0,506** |  |  |  | 0,321 |
|  | **≥70** |  |  |  |  | *0,114* |  | 0,078 | **0,454** |  |  |  | 0,255 |
| **≥70** | **<70** |  |  |  |  | 0,095 |  | 0,056 | **0,548** |  | 0,043 |  | 0,340 |
|  | **≥70** |  |  |  |  | 0,091 |  | 0,070 | **0,520** |  |  |  | 0,306 |
| **FEMALE, stPP** | | | | | | | | | | | | | |
| **AGE** | **HR** | smoking | coffee consump-tion | snoring | lack of phys. activity | obesity | family history | diabetes | hyper-tension | CVD | chronic kidney disease | hyper-choleste-rinaemia | R^2^ |
| **<40** | **<70** |  |  |  |  |  |  |  |  |  |  |  | ns |
|  | **≥70** |  |  |  |  | 0,095 |  |  | *0,171* |  |  |  | 0,050 |
| **40-49** | **<70** |  |  |  |  |  |  |  | **0,265** |  |  |  | 0,092 |
|  | **≥70** |  |  |  |  |  |  |  | *0,163* |  |  |  | 0,036 |
| **50-59** | **<70** |  |  |  |  |  |  |  | **0,252** |  |  |  | 0,070 |
|  | **≥70** |  |  |  |  |  |  | 0,075 | *0,162* |  |  |  | 0,036 |
| **60-69** | **<70** |  |  |  |  | 0,074 |  | *0,116* | **0,260** | 0,068 | 0,081 |  | 0,122 |
|  | **≥70** |  |  |  |  |  |  |  | *0,176* | 0,060 |  |  | 0,046 |
| **≥70** | **<70** |  |  |  |  |  |  |  | **0,302** | 0,065 |  |  | 0,106 |
|  | **≥70** |  |  |  |  |  |  | 0,074 | **0,213** |  |  |  | 0,055 |

| **MALE, elPP** | | | | | | | | | | | | | |
| --- | --- | --- | --- | --- | --- | --- | --- | --- | --- | --- | --- | --- | --- |
| **AGE** | **HR** | smoking | coffee consump-tion | snoring | lack of phys. activity | obesity | family history | diabetes | hyper-tension | CVD | chronic kidney disease | hyper-choleste-rinaemia | R^2^ |
| **<40** | **<70** |  |  |  | -0,118 |  |  |  | **0,364** |  |  | *-0,104* | 0,168 |
|  | **≥70** |  |  |  |  | *0,126* |  |  | **0,233** |  |  | -0,060 | 0,079 |
| **40-49** | **<70** |  |  |  |  | *0,160* |  |  | **0,204** |  |  |  | 0,092 |
|  | **≥70** |  |  |  |  | *0,192* |  | 0,060 | *0,182* |  |  | -0,064 | 0,083 |
| **50-59** | **<70** |  |  |  |  | *0,139* |  | *0,112* | **0,313** |  |  |  | 0,149 |
|  | **≥70** | 0,067 |  |  | 0,07 | *0,168* |  |  | **0,245** |  |  |  | 0,113 |
| **60-69** | **<70** |  |  |  |  | *0,117* |  |  | **0,453** |  |  |  | 0,263 |
|  | **≥70** |  |  |  |  | *0,141* |  |  | **0,391** |  |  |  | 0,200 |
| **≥70** | **<70** |  |  |  |  | 0,073 |  | *0,108* | **0,521** |  |  |  | 0,313 |
|  | **≥70** |  |  |  |  | *0,111* |  |  | **0,475** |  |  |  | 0,269 |

| **MALE, stPP** | | | | | | | | | | | | | |
| --- | --- | --- | --- | --- | --- | --- | --- | --- | --- | --- | --- | --- | --- |
| **AGE** | **HR** | smoking | coffee consumption | snoring | lack of phys. activity | obesity | family history | diabetes | hyper-tension | CVD | chronic kidney disease | hyper-choleste-rinaemia | R^2^ |
| **<40** | **<70** |  |  |  |  |  |  |  | *0,138* |  |  |  | 0,040 |
|  | **≥70** |  |  |  |  |  |  |  | *0,116* |  |  |  | 0,025 |
| **40-49** | **<70** |  |  |  |  |  |  | 0,086 | *0,145* |  |  |  | 0,036 |
|  | **≥70** |  |  |  |  |  |  |  | 0,097 |  |  |  | 0,015 |
| **50-59** | **<70** |  |  |  |  |  |  |  | *0,171* |  |  |  | 0,043 |
|  | **≥70** |  |  |  |  |  |  | 0,077 | *0,127* |  |  |  | 0,027 |
| **60-69** | **<70** |  |  |  |  |  |  | *0,135* | **0,241** |  |  |  | 0,090 |
|  | **≥70** |  |  |  |  |  |  | *0,101* | *0,160* |  |  |  | 0,043 |
| **≥70** | **<70** |  |  |  |  |  |  | 0,073 | **0,293** |  |  |  | 0,103 |
|  | **≥70** |  |  |  |  |  |  |  | **0,213** |  |  |  | 0,052 |

CVD: cardiovascular disease; elPP: elastic pulse pressure; HR: heart rate; lack of phys. activity: lack of regular physical activity; R^2^: coefficient of determination; stPP: stiffening pulse pressure. Values are beta coefficients of multiple regression analyses. Bold: clinically significant strong predictor; Bold in italics: clinically significant moderate predictor; unformatted: statistically significant predictor.
