# Supplementary material for: Associated factors of elastic and stiffening components of pulse pressure based on 45 517 database of 24 h ambulatory blood pressure monitoring
Source: J Hum Hypertens. 2026 Aug 26;40(11):855–64. doi: 10.1038/s41371-026-01210-0 (PMC13747817; doi:10.1038/s41371-026-01210-0)
Supplement: Supplementary file 3 [file 41371_2026_1210_MOESM3_ESM.docx]

**Supplementary table 3.** The explanatory variables of elPP and stPP in different sexes and heart rates in the detailed different age categories in subjects without antihypertensive treatment.

| **FEMALE, elPP** | | | | | | | | | | | | | |
| --- | --- | --- | --- | --- | --- | --- | --- | --- | --- | --- | --- | --- | --- |
| **AGE** | **HR** | smoking | coffee consump-tion | snoring | lack of phys. activity | obesity | family history | diabetes | hyper-tension | CVD | chronic kidney disease | hyper-choleste-rinaemia | R^2^ |
| **<40** | **<70** |  |  |  |  |  |  |  |  | NA |  |  | ns |
|  | **≥70** |  |  |  |  | **0,305** |  |  | **0,215** |  | NA |  | 0,190 |
| **40-49** | **<70** |  |  |  |  | *0,199* |  |  | **0,259** |  |  |  | 0,141 |
|  | **≥70** |  |  |  |  | **0,302** |  |  | **0,253** |  |  |  | 0,198 |
| **50-59** | **<70** |  |  |  |  | *0,181* |  |  | **0,407** |  |  |  | 0,271 |
|  | **≥70** |  |  |  |  | **0,213** |  |  | **0,287** |  |  |  | 0,165 |
| **60-69** | **<70** |  |  |  |  |  |  |  | **0,493** |  |  |  | 0,263 |
|  | **≥70** |  |  |  |  |  |  |  | **0,401** |  |  |  | 0,223 |
| **≥70** | **<70** |  |  |  |  |  |  |  | **0,530** |  |  |  | 0,336 |
|  | **≥70** |  |  |  |  |  |  |  | **0,541** |  |  |  | 0,364 |
| **FEMALE, stPP** | | | | | | | | | | | | | |
| **AGE** | **HR** | smoking | coffee consump-tion | snoring | lack of phys. activity | obesity | family history | diabetes | hyper-tension | CVD | chronic kidney disease | hyper-choleste-rinaemia | R^2^ |
| **<40** | **<70** |  |  |  |  |  |  |  |  | NA |  |  | ns |
|  | **≥70** |  |  |  |  | 0,097 |  |  | *0,168* |  | NA |  | 0,048 |
| **40-49** | **<70** |  |  |  |  |  |  |  | **0,231** |  |  |  | 0,086 |
|  | **≥70** |  |  |  |  |  |  |  | *0,153* |  |  |  | 0,036 |
| **50-59** | **<70** |  |  |  |  |  |  |  | **0,232** |  |  |  | 0,061 |
|  | **≥70** |  |  |  |  |  |  |  | *0,146* |  |  |  | 0,026 |
| **60-69** | **<70** |  |  |  |  |  |  |  | **0,211** |  |  |  | 0,066 |
|  | **≥70** |  |  |  |  |  |  |  | *0,155* |  |  |  | 0,044 |
| **≥70** | **<70** |  |  |  |  |  |  |  | **0,353** |  |  |  | 0,173 |
|  | **≥70** |  |  |  |  |  |  |  | **0,221** |  |  |  | 0,071 |

| **MALE, elPP** | | | | | | | | | | | | | |
| --- | --- | --- | --- | --- | --- | --- | --- | --- | --- | --- | --- | --- | --- |
| **AGE** | **HR** | smoking | coffee consump-tion | snoring | lack of phys. activity | obesity | family history | diabetes | hyper-tension | CVD | chronic kidney disease | hyper-choleste-rinaemia | R^2^ |
| **<40** | **<70** |  |  |  |  |  |  |  | **0,336** |  | NA |  | 0,145 |
|  | **≥70** |  |  |  |  | *0,131* |  |  | **0,230** |  |  |  | 0,083 |
| **40-49** | **<70** |  |  |  |  | *0,186* |  |  | **0,201** |  |  |  | 0,099 |
|  | **≥70** |  |  |  |  | *0,147* |  |  | *0,161* |  |  |  | 0,058 |
| **50-59** | **<70** |  |  |  |  |  |  |  | **0,207** |  |  |  | 0,126 |
|  | **≥70** |  |  |  |  | **0,236** |  | *0,104* | **0,230** |  |  |  | 0,147 |
| **60-69** | **<70** |  |  |  |  | *0,176* |  |  | **0,388** |  | NA |  | 0,209 |
|  | **≥70** |  |  |  |  |  | *0,162* |  | **0,337** |  |  |  | 0,161 |
| **≥70** | **<70** |  |  |  |  |  |  |  | **0,515** |  |  |  | 0,316 |
|  | **≥70** |  |  |  |  |  |  |  | **0,471** |  |  |  | 0,284 |

| **MALE, stPP** | | | | | | | | | | | | | |
| --- | --- | --- | --- | --- | --- | --- | --- | --- | --- | --- | --- | --- | --- |
| **AGE** | **HR** | smoking | coffee consumption | snoring | lack of phys. activity | obesity | family history | diabetes | hyper-tension | CVD | chronic kidney disease | hyper-choleste-rinaemia | R^2^ |
| **<40** | **<70** |  |  |  |  |  |  |  |  |  | NA |  | ns |
|  | **≥70** |  |  |  |  |  |  |  | *0,108* |  |  |  | 0,023 |
| **40-49** | **<70** |  |  |  |  |  |  |  | *0,169* |  |  |  | 0,053 |
|  | **≥70** |  |  |  |  |  |  |  |  |  |  |  | ns |
| **50-59** | **<70** |  |  |  |  |  |  |  | *0,187* |  |  |  | 0,053 |
|  | **≥70** |  |  |  |  |  |  |  |  |  |  |  | ns |
| **60-69** | **<70** |  |  |  |  |  |  |  | 0,131 |  | NA |  | 0,072 |
|  | **≥70** |  |  |  |  |  |  |  |  |  |  |  | ns |
| **≥70** | **<70** |  |  |  |  |  |  |  |  |  |  |  | ns |
|  | **≥70** |  |  |  |  |  |  |  |  |  |  |  | ns |

CVD: cardiovascular disease; elPP: elastic pulse pressure; HR: heart rate; lack of phys. activity: lack of regular physical activity; R^2^: coefficient of determination; stPP: stiffening pulse pressure. Values are beta coefficients of multiple regression analyses. Bold: clinically significant strong predictor; Bold in italics: clinically significant moderate predictor; unformatted: statistically significant predictor. NA: not included in the model, cannot be computed.
