# Supplementary material for: Associated factors of elastic and stiffening components of pulse pressure based on 45 517 database of 24 h ambulatory blood pressure monitoring
Source: J Hum Hypertens. 2026 Aug 26;40(11):855–64. doi: 10.1038/s41371-026-01210-0 (PMC13747817; doi:10.1038/s41371-026-01210-0)
Supplement: Supplementary file 4 [file 41371_2026_1210_MOESM4_ESM.docx]

**Supplementary table 4.** The explanatory variables of elPP and stPP in different sexes and heart rates in the detailed different age categories in subjects with ongoing antihypertensive treatment.

| **FEMALE, elPP** | | | | | | | | | | | | | | | |
| --- | --- | --- | --- | --- | --- | --- | --- | --- | --- | --- | --- | --- | --- | --- | --- |
| **AGE** | **HR** | | smoking | coffee consump-tion | snoring | lack of phys. activity | obesity | family history | diabetes | hyper-tension | CVD | chronic kidney disease | hyper-choleste-rinaemia | BB | R^2^ |
| **<40** | **<70** | |  |  |  |  | **0,280** |  |  |  |  |  |  |  | 0,215 |
|  | **≥70** | |  |  |  |  | **0,351** |  |  | **0,238** |  |  |  |  | 0,201 |
| **40-49** | **<70** | |  |  |  |  | **0,265** |  |  | **0,293** |  |  |  |  | 0,210 |
|  | **≥70** | |  |  | 0,080 |  | **0,246** |  |  | **0,254** |  |  |  |  | 0,167 |
| **50-59** | **<70** | |  |  |  |  | **0,230** |  |  | **0,410** |  |  |  |  | 0,271 |
|  | **≥70** | |  |  |  | 0,062 | **0,215** |  |  | **0,331** |  |  | -0,062 |  | 0,219 |
| **60-69** | **<70** | |  |  |  |  | *0,135* |  | 0,089 | **0,510** |  |  |  |  | 0,332 |
|  | **≥70** | |  |  |  |  | *0,111* |  | 0,072 | **0,467** |  |  |  |  | 0,265 |
| **≥70** | **<70** | |  |  |  |  | *0,102* |  | 0,054 | **0,548** |  |  |  |  | 0,344 |
|  | **≥70** | |  |  |  |  | 0,096 |  | 0,069 | **0,518** |  |  |  |  | 0,303 |
|  | | **FEMALE, stPP** | | | | | | | | | | | | | |
| **AGE** | **HR** | | smoking | coffee consump-tion | snoring | lack of phys. activity | obesity | family history | diabetes | hyper-tension | CVD | chronic kidney disease | hyper-choleste-rinaemia | BB | R^2^ |
| **<40** | **<70** | |  |  |  |  |  |  |  | **0,265** |  |  |  |  | 0,177 |
|  | **≥70** | |  |  |  |  |  |  |  | *0,161* |  |  |  |  | 0,060 |
| **40-49** | **<70** | |  |  |  |  |  |  |  | **0,292** |  |  |  |  | 0,109 |
|  | **≥70** | |  |  |  |  |  |  |  | *0,173* |  |  |  |  | 0,041 |
| **50-59** | **<70** | |  |  |  |  |  |  |  | **0,263** |  |  |  |  | 0,080 |
|  | **≥70** | |  |  |  |  |  |  | 0,083 | *0,174* |  |  |  |  | 0,042 |
| **60-69** | **<70** | |  |  |  |  | 0,075 |  | *0,120* | **0,270** | 0,066 | 0,089 |  |  | 0,131 |
|  | **≥70** | |  |  |  |  |  |  |  | *0,184* |  |  |  |  | 0,051 |
| **≥70** | **<70** | |  |  |  |  |  |  |  | **0,303** | 0,060 |  |  |  | 0,105 |
|  | **≥70** | |  |  |  |  |  |  | 0,075 | **0,214** |  |  |  |  | 0,056 |

| **MALE, elPP** | | | | | | | | | | | | | | |
| --- | --- | --- | --- | --- | --- | --- | --- | --- | --- | --- | --- | --- | --- | --- |
| **AGE** | **HR** | smoking | coffee consump-tion | snoring | lack of phys. activity | obesity | family history | diabetes | hyper-tension | CVD | chronic kidney disease | hyper-choleste-rinaemia | BB | R^2^ |
| **<40** | **<70** |  |  |  |  |  |  |  | **0,387** |  |  |  |  | 0,216 |
|  | **≥70** |  |  |  |  | *0,120* |  |  | **0,237** |  |  |  |  | 0,084 |
| **40-49** | **<70** |  |  |  |  | *0,136* |  |  | **0,203** |  |  |  |  | 0,107 |
|  | **≥70** |  |  |  |  | **0,210** |  |  | *0,197* |  |  |  |  | 0,099 |
| **50-59** | **<70** |  |  |  |  | *0,137* |  | *0,111* | **0,348** |  |  |  |  | 0,168 |
|  | **≥70** | 0,075 |  |  | 0,074 | *0,140* |  | *0,077* | **0,252** |  |  |  |  | 0,110 |
| **60-69** | **<70** |  |  |  |  | *0,107* |  | *0,135* | **0,463** |  |  |  |  | 0,275 |
|  | **≥70** |  |  |  |  | *0,139* |  | 0,095 | **0,405** |  |  |  |  | 0,216 |
| **≥70** | **<70** |  |  |  |  | 0,071 |  | *0,105* | **0,524** |  |  |  |  | 0,316 |
|  | **≥70** |  |  |  |  | *0,127* |  |  | **0,474** |  |  |  |  | 0,278 |

| **MALE, stPP** | | | | | | | | | | | | | | |
| --- | --- | --- | --- | --- | --- | --- | --- | --- | --- | --- | --- | --- | --- | --- |
| **AGE** | **HR** | smoking | coffee consumption | snoring | lack of phys. activity | obesity | family history | diabetes | hyper-tension | CVD | chronic kidney disease | hyper-choleste-rinaemia | BB | R^2^ |
| **<40** | **<70** |  |  |  | *-0,178* |  |  |  | *0,181* |  |  |  |  | 0,092 |
|  | **≥70** |  |  |  |  |  |  |  | *0,124* |  |  |  |  | 0,035 |
| **40-49** | **<70** |  |  |  |  |  |  |  | *0,128* |  |  |  |  | 0,035 |
|  | **≥70** |  |  |  |  |  |  |  | *0,112* |  |  |  |  | 0,020 |
| **50-59** | **<70** |  |  |  |  |  |  |  | *0,178* |  |  |  |  | 0,048 |
|  | **≥70** |  |  |  |  |  |  | 0,079 | *0,137* |  |  |  |  | 0,035 |
| **60-69** | **<70** |  |  |  |  |  |  | *0,127* | **0,258** |  |  |  |  | 0,096 |
|  | **≥70** |  |  |  |  |  |  | 0,088 | *0,176* |  |  |  |  | 0,047 |
| **≥70** | **<70** |  |  |  |  |  |  | 0,073 | **0,309** |  |  |  |  | 0,114 |
|  | **≥70** |  |  |  |  |  |  |  | **0,219** |  |  |  |  | 0,056 |

CVD: cardiovascular disease; elPP: elastic pulse pressure; HR: heart rate; lack of phys. activity: lack of regular physical activity; R^2^: coefficient of determination; stPP: stiffening pulse pressure; BB: beta blockers. Values are beta coefficients of multiple regression analyses. Bold: clinically significant strong predictor; Bold in italics: clinically significant moderate predictor; unformatted: statistically significant predictor. NA: not included in the model, cannot be computed.
