# Supplementary material for: Associated factors of elastic and stiffening components of pulse pressure based on 45 517 database of 24 h ambulatory blood pressure monitoring
Source: J Hum Hypertens. 2026 Aug 26;40(11):855–64. doi: 10.1038/s41371-026-01210-0 (PMC13747817; doi:10.1038/s41371-026-01210-0)
Supplement: Supplementary file 5 [file 41371_2026_1210_MOESM5_ESM.docx]

**Supplementary Figure 1.** elPP (A) and stPP (B) by sex and age in the no antihypertensive treatment sample

A

elPP mean (mmHg, mean)

| Age groups (years) | female | male |
| --- | --- | --- |
| <40 | 43.84 | 49.54 |
| 40-49 | 42.48 | 44.1 |
| 50-59 | 43.14 | 43.79 |
| 60-69 | 45.54 | 45.45 |
| ≥70 | 49.1 | 47.98 |

B

stPP mean (mmHg, mean)

| Age groups (years) | female | male |
| --- | --- | --- |
| <40 | 4.56 | 5.58 |
| 40-49 | 6.31 | 5.99 |
| 50-59 | 7.63 | 7.4 |
| 60-69 | 9.19 | 9.34 |
| ≥70 | 10.54 | 10.71 |
