# Supplementary material for: Associated factors of elastic and stiffening components of pulse pressure based on 45 517 database of 24 h ambulatory blood pressure monitoring
Source: J Hum Hypertens. 2026 Aug 26;40(11):855–64. doi: 10.1038/s41371-026-01210-0 (PMC13747817; doi:10.1038/s41371-026-01210-0)
Supplement: Supplementary file 6 [file 41371_2026_1210_MOESM6_ESM.docx]

**Supplementary Figure 2.** elPP (A) and stPP (B) by sex and age in the antihypertensive treatment sample

A

elPP mean (mmHg, mean)

| Age groups (years) | female | male |
| --- | --- | --- |
| <40 | 44.25 | 49.5 |
| 40-49 | 42.84 | 45.03 |
| 50-59 | 43.83 | 44.47 |
| 60-69 | 46.69 | 46.21 |
| ≥70 | 49.89 | 48.64 |

B

stPP mean (mmHg)

| Age groups (years) | female | male |
| --- | --- | --- |
| <40 | 4.89 | 6.14 |
| 40-49 | 6.67 | 6.34 |
| 50-59 | 8.14 | 7.83 |
| 60-69 | 10.18 | 10.28 |
| ≥70 | 12.09 | 12.42 |
